# The Dresden Dataset for 4D Reconstruction of Non-Rigid Abdominal Surgical Scenes

**DOI:** 10.1038/s41597-026-08289-7

**Published:** 2026-09-21

**Authors:** Reuben Docea, Rayan Younis, Yonghao Long, Maxime Fleury, Jinjing Xu, Chenyang Li, André Schulze, Ann Wierick, Johannes Bender, Micha Pfeiffer, Qi Dou, Martin Wagner, Stefanie Speidel

**Affiliations:** 1 Department of Translational Surgical Oncology, National Center for Tumor Diseases (NCT), NCT/UCC Dresden, a partnership between DKFZ, Faculty of Medicine and University Hospital Carl Gustav Carus, TUD Dresden University of Technology, and Helmholtz-Zentrum Dresden-Rossendorf (HZDR), Dresden, Germany; 2 Department of Translational Surgical Oncology, NCT/UCC Dresden, Faculty of Medicine and University Hospital Carl Gustav Carus, TUD Dresden University of Technology, Fetscherstraße 74, 01307 Dresden, Germany; 3 Department for Visceral, Thoracic and Vascular Surgery, Faculty of Medicine and University Hospital Carl Gustav Carus, Technische Universität Dresden, Dresden, Germany; 4 Centre for Tactile Internet with Human-in-the-Loop (CeTI), Technical University Dresden, Dresden, Germany; 5 Department of Computer Science and Engineering, The Chinese University of Hong Kong, Hong Kong, China

## Abstract

The **D4D** Dataset provides paired endoscopic video and high-quality structured-light geometry for evaluating 3D reconstruction of deforming abdominal soft tissue in realistic surgical conditions. Data were acquired from six porcine cadavers using a da Vinci Xi stereo endoscope and a Zivid structured-light camera, registered via optical tracking and manually curated iterative alignment methods. Three session types (whole deformations, incremental deformations, and moved-camera sessions) probe algorithm robustness to non-rigid motion, deformation magnitude, and out-of-view updates. Each clip provides rectified stereo images, per-frame instrument masks, stereo depth, start/end structured-light point clouds, curated camera poses and camera intrinsics. In postprocessing, ICP and semi-automatic registration techniques are used to register data, and instrument masks are created. The dataset enables quantitative geometric evaluation in both visible and occluded regions, alongside photometric view-synthesis baselines. Comprising over 300,000 frames and 369 point clouds across 98 curated sessions, this resource can serve as a comprehensive benchmark for developing and evaluating non-rigid SLAM, 4D reconstruction, and depth estimation methods.

## Background & Summary

The ability to accurately reconstruct deforming soft tissue scenes is foundational to many computer-aided innovations in minimally invasive surgery (MIS). These methods are essential in particular for Image Guidance Systems (IGS), which are used for intraoperative navigation. Here, they provide a complete view of the surgical scene to improve spatial understanding, thereby supporting enhanced decision-making and safety^1–3^. This improved guidance enables more precise instrument control and represents a key step towards robotic surgery automation^1,4,5^. For patients, these capabilities could lead to more effective interventions and may reduce the need for invasive follow-up procedures^6^. Beyond the operating room, such reconstruction techniques are also highly valuable for creating realistic simulators for surgical education, training, and rehearsal.

However, IGS built on these principles have only seen scarce up-take for the case of abdominal surgery^7^. A primary reason for this is that the abdominal environment is non-rigid and continually subject to change. Designing a navigation system for such conditions is a considerable endeavour which requires a thorough understanding of system accuracy.

Soft tissue navigation typically relies upon the registration of preoperative data (such as CT or MRI scans) with intraoperative data, often consisting of a 3D map of the tissue surface^8–10^. These 3D maps, however, require time to create, and the methods to do so are often designed with the assumption that the scene is rigid. This means that as soon as the shape of the tissue changes, through manipulation from the surgeon or otherwise, it must be re-mapped for any subsequent registration to be accurate, which takes a substantial amount of time.

For this scenario, where tissue deforms even out of view^11,12^, a non-rigid mapping method is required. Such a method must realistically update previously mapped areas, which are no longer visible, to fit current observations. While there are works which go in this direction, there is no dataset available at present that serves to evaluate this capability. A first method which makes inroads into this problem is that of MIS-SLAM^13^, which utilises an embedded deformation graph^14^ and a heuristics-based optimisation objective to update a global map from current observations. In a recent work^12^, we similarly attempted, using a GNN, to predict the positions of points which were seen but moved out of view, based on currently visible points.

In recent years, there have been many efforts at performing 3D reconstruction of non-rigid structures^15,16^. Of particular interest, these efforts span into the surgical domain, where soft tissue is concerned^3^. Among such efforts are firstly some that pertain to single view-point 4D reconstructions, learning the deformations of specific scenes^1,2,17–22^. Besides these, efforts to this end exist in the context of non-rigid SLAM^13,23^. However, existing datasets (such as SCARED^24^, SERV-CT^25^, EndoMapper^11^ and StereoMIS^26^) do not possess endoscopic video paired with high quality ground truth geometric data which can be used to evaluate 3D reconstruction in deforming soft tissue contexts. The authors of EndoNeRF remark, “Due to clinical regulation in practice, it is impossible to collect ground truth depth for numerical evaluation on 3D structures”^1^. This leads to the formerly mentioned 4D reconstruction methods evaluating with reference almost exclusively to photometric errors, such as: PSNR (Peak Signal to Noise Ratio)^27^, SSIM (Structural Similarity Index Measure)^28^ and LPIPS (Learned Perceptual Image Patch Similarity)^29^.

The **D4D Dataset** fills a crucial gap by providing the first dataset containing endoscopic video paired with reference geometric data, acquired using a structured-light camera (SLC) to capture the initial and final tissue states in controlled manipulations of porcine cadaver abdominal soft tissue. The dataset comprises three types of sessions:

(i) **Whole deformation**: complete manipulations (pushing or pulling).
(ii) **Incremental deformation**: a manipulation divided into smaller increments, allowing detailed analysis of the tissue’s deformation process.
(iii) **Moved camera**: an action performed in two halves - first with a static camera, then after the camera has been repositioned. This setup enables evaluation of how accurately structures that deform out of view can be reconstructed realistically.

Because the endoscope and SLC occupy distinct viewpoints, the dataset facilitates quantitative evaluation of geometric reconstruction accuracy in both visible and occluded regions in 3D space. Consequently, it provides a valuable resource for developing and assessing non-rigid SLAM and reconstruction algorithms under realistic, deformable surgical conditions. An accompanying project page can be found at: https://reubendocea.github.io/d4d/.

## Methods

### Data Collection

#### Experimental Setup

**Fig. 1:**
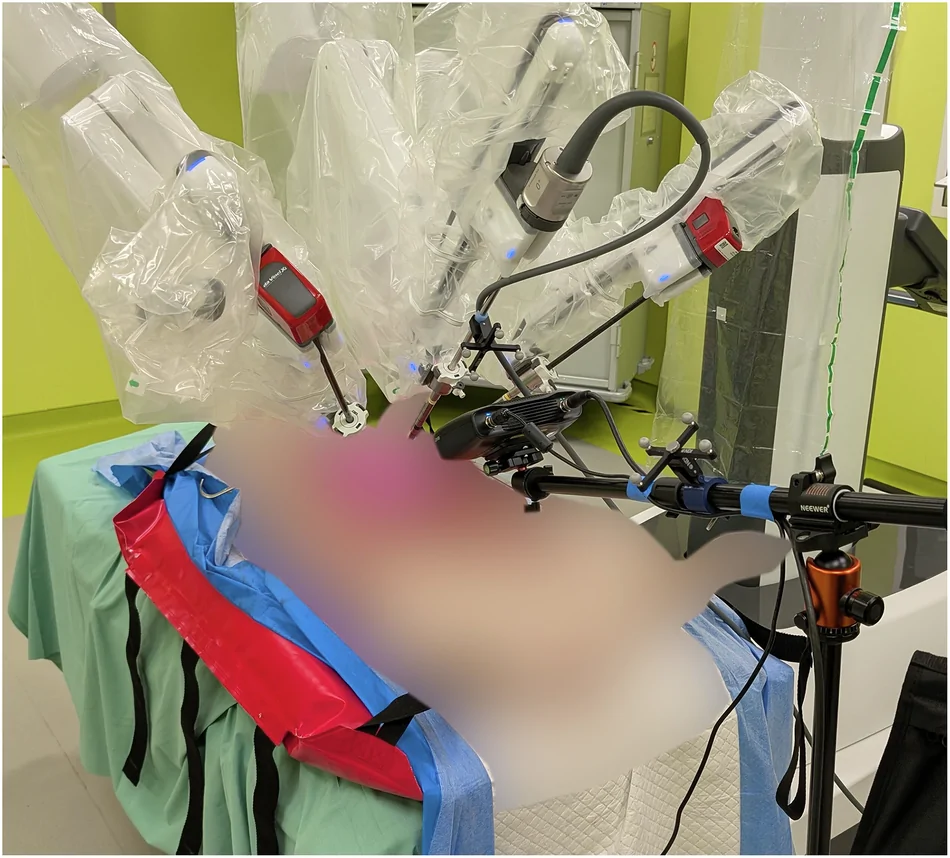
Experimental setup showing porcine cadaver in supine position for open surgery with *da Vinci*. The *Zivid* SLC is visible in the foreground, with optical markers attached to the boom supporting the SLC and the endoscope.

Between February 2025 and September 2025, data were collected using six porcine cadavers at the Experimental Operating Room of the *National Center for Tumor Diseases* in *Dresden, Germany*. All procedures were carried out with a *da Vinci Xi* surgical robot (Intuitive Inc., Sunnyvale, USA) equipped with a stereo endoscope, using either a 30° or a 0° camera tip. Both left and right endoscopic images were recorded at a resolution of 894 × 714 pixels. To capture complementary geometric information, a Zivid 2+ M60 (Zivid AS, Oslo, Norway) was employed as the SLC. Both the endoscope and the SLC were rigidly fitted with optical fiducial markers, whose poses were tracked in real time by a Polaris Vega optical tracking system (Northern Digital Inc., Ontario, Canada). This setup enabled accurate registration between the visual and geometric data streams (Fig. 1). The manufacturer-specified accuracies of the sensors are as follows. The Polaris Vega has a volumetric tracking accuracy of 0.12 mm RMS^30^. At its 600 mm focus distance, the Zivid 2+ M60 has a point precision of 80 μm (one standard deviation) and a dimension trueness error below 0.25% across its 350–900 mm optimal working range^31^. The endoscope, SLC and Polaris data streams were recorded simultaneously on a workstation with Robot Operating System 2 (ROS2^32^), as *rosbags*. Synchronisation was achieved via timestamping of all messages using the ROS2 system clock, ensuring temporal alignment across data streams. This timestamping does not compensate for communication or driver-scheduling delays, so the tracker-derived poses are only approximate, and the curated camera-to-SLC pose (Methods) should be preferred for the static states. The tracker-derived poses are retained as the only pose source for the moving portion of moved-camera clips, where no SLC capture is available. On some of the data collection days, the relevant anatomical structures of the pig which are relevant to this study were operated on in the prior procedures.

#### Experimental Protocol

**Fig. 2:**
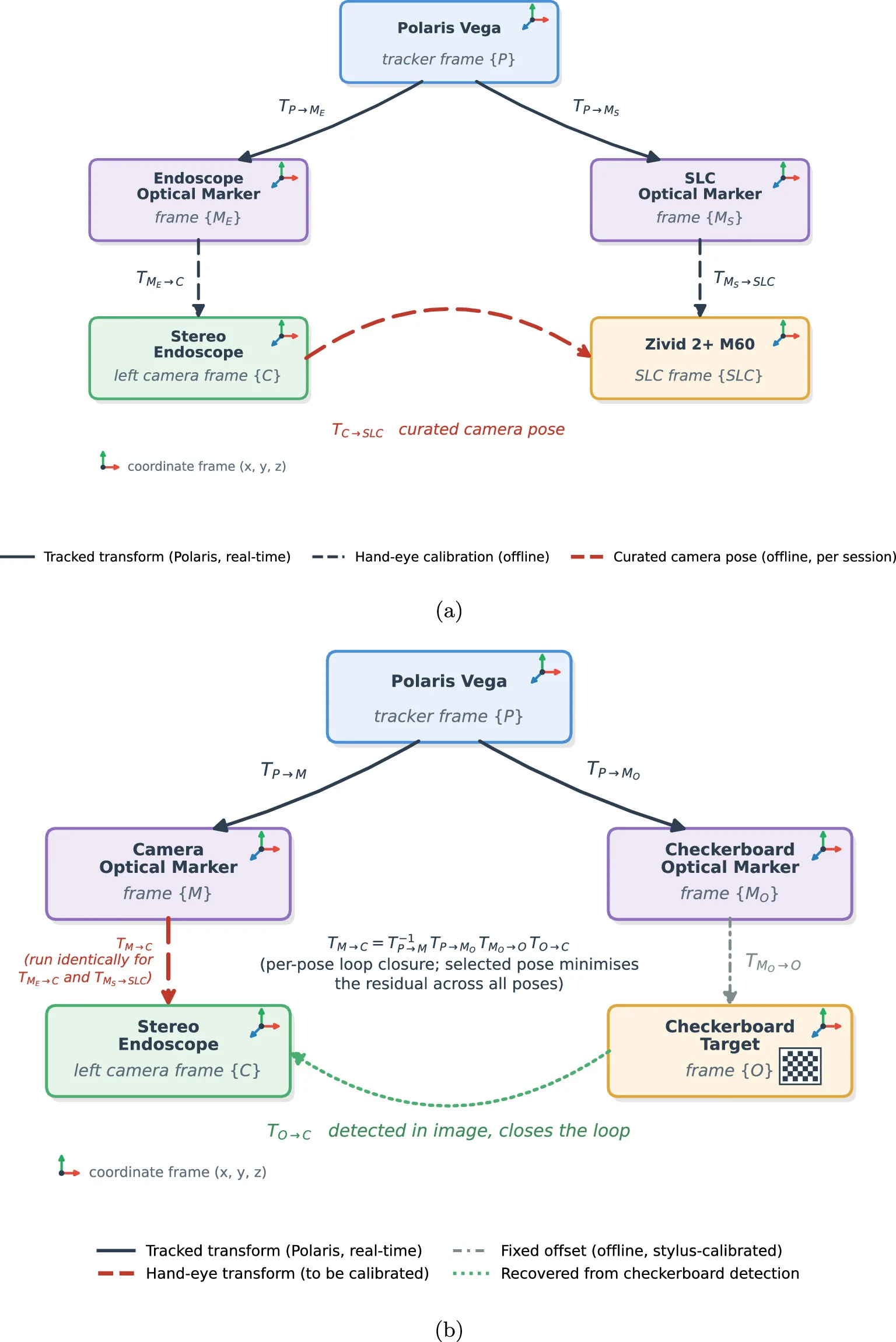
Coordinate frames and rigid transforms of the acquisition setup. (**a**) The Polaris Vega tracker ({*P*}) tracks the optical markers on the endoscope ({*M*_*E*_}) and the SLC ({*M*_*S*_}); hand-eye calibration fixes ${T}_{{M}_{E}\to C}$ and ${T}_{{M}_{S}\to SLC}$, and the curated pose *T*_*C*→*SLC*_ aligns the stereo reconstructions with the SLC point clouds. (**b**) Hand-eye calibration setup, shown generically as *T*_*M*→*C*_: the checkerboard is tracked indirectly via its own marker ({*M*_*O*_}), related to its origin {*O*} by a fixed, stylus-derived offset ${T}_{{M}_{O}\to O}$. Per pose, the image-detected pose *T*_*O*→*C*_ closes the loop ${T}_{M\to C}={T}_{P\to M}^{-1}{T}_{P\to {M}_{O}}{T}_{{M}_{O}\to O}{T}_{O\to C}$, whose residual is minimised across all poses to select the calibration result.

The stereo endoscope first underwent standard camera calibration following Zhang’s method^33^, and hand-eye calibrations were performed to relate the poses of both the endoscope and the SLC to their respective tracking markers. An overview of the coordinate frames and the rigid transforms relating them is given in Fig. 2. Both endoscope cameras and the SLC were modelled with the Brown-Conrady radial-tangential distortion model^34^ (the plumb_bob model in ROS, equivalent to OpenCV’s standard distortion coefficients), parameterised by five coefficients (*k*_1_, *k*_2_, *p*_1_, *p*_2_, *k*_3_). A 3D point (*X*, *Y*, *Z*) in the camera frame is normalised as *x* = *X*/*Z*, *y* = *Y*/*Z*, with *r*^2^ = *x*^2^ + *y*^2^, and mapped to distorted normalised coordinates

1 $${x}_{d}=x\,(1+{k}_{1}{r}^{2}+{k}_{2}{r}^{4}+{k}_{3}{r}^{6})+2{p}_{1}xy+{p}_{2}({r}^{2}+2{x}^{2}),$$

2 $${y}_{d}=y\,(1+{k}_{1}{r}^{2}+{k}_{2}{r}^{4}+{k}_{3}{r}^{6})+{p}_{1}({r}^{2}+2{y}^{2})+2{p}_{2}xy,$$

which project to pixel coordinates *u* = *f*_*x*_*x*_*d*_ + *c*_*x*_ and *v* = *f*_*y*_*y*_*d*_ + *c*_*y*_, where (*f*_*x*_, *f*_*y*_) and (*c*_*x*_, *c*_*y*_) are the focal length and principal point. The per-camera intrinsic and distortion parameters are provided in the camera_info files. The calibration target and the calibration images used for one representative session are provided in the accompanying code repository. The intrinsic stereo calibration achieved a sub-pixel root-mean-square reprojection error of approximately 0.6 pixels.

This hand-eye calibration used a planar checkerboard target that was tracked indirectly via a separate optical marker rigidly attached to it, frame {M_O_}, since the checkerboard’s own origin frame {O} (defined by its inner corner grid) could not itself be tracked directly. The fixed transform ${T}_{{{\rm{M}}}_{{\rm{O}}}\to {\rm{O}}}$ between these two frames was derived once, offline, by using a calibrated, optically tracked stylus to probe the positions of the checkerboard’s inner corners relative to its optical marker, from which the checkerboard’s origin and axes – and hence ${T}_{{{\rm{M}}}_{{\rm{O}}}\to {\rm{O}}}$ – were computed. At each calibration pose, three transforms were recorded: the tracked pose of the camera marker in the tracker frame *T*_P→M_, the tracked pose of the checkerboard’s optical marker in the tracker frame ${T}_{{\rm{P}}\to {{\rm{M}}}_{{\rm{O}}}}$, which combined with the fixed offset ${T}_{{{\rm{M}}}_{{\rm{O}}}\to {\rm{O}}}$ gives the pose of the checkerboard itself, ${T}_{{\rm{P}}\to {\rm{O}}}={T}_{{\rm{P}}\to {{\rm{M}}}_{{\rm{O}}}}\,{T}_{{{\rm{M}}}_{{\rm{O}}}\to {\rm{O}}}$, and the pose of the checkerboard in the camera frame *T*_O→C_ obtained by detecting the board in the image. As the target was tracked (via its marker) in addition to the camera, the marker-to-camera transform was obtained directly for each pose as ${T}_{{\rm{M}}\to {\rm{C}}}={T}_{{\rm{P}}\to {\rm{M}}}^{-1}\,{T}_{{\rm{P}}\to {\rm{O}}}\,{T}_{{\rm{O}}\to {\rm{C}}}$, without solving the usual *AX* = *XB* hand-eye system. Several poses were recorded, and the final transform was selected as the per-pose estimate that minimised the median pose-consistency error across the remaining poses, where closing the transform loop for each comparison pose yields a residual whose translation magnitude and rotation angle define a translation and a rotation error. For the calibrations performed in this study, the residual translation of the transform chain that loops from the Polaris frame back to the Polaris frame was typically below 5 mm for the SLC and around 12 mm for the endoscope. These residual magnitudes are consistent with the approximate tracked-pose alignment noted below, which the per-clip pose curation subsequently refines.

Following calibration and conversion to open surgery, the data collection began. Room lighting was turned off at the beginning of data collection to enhance SNR of SLC and to better emulate lighting conditions of MIS. The room lighting remained off for the entire session. Aside from the structured-light projection emitted by the SLC during point-cloud capture, the endoscope cold-light source was the only illumination of the scene and was held at a fixed intensity throughout. It was switched off only during the SLC captures, as described below.

**Fig. 3:**
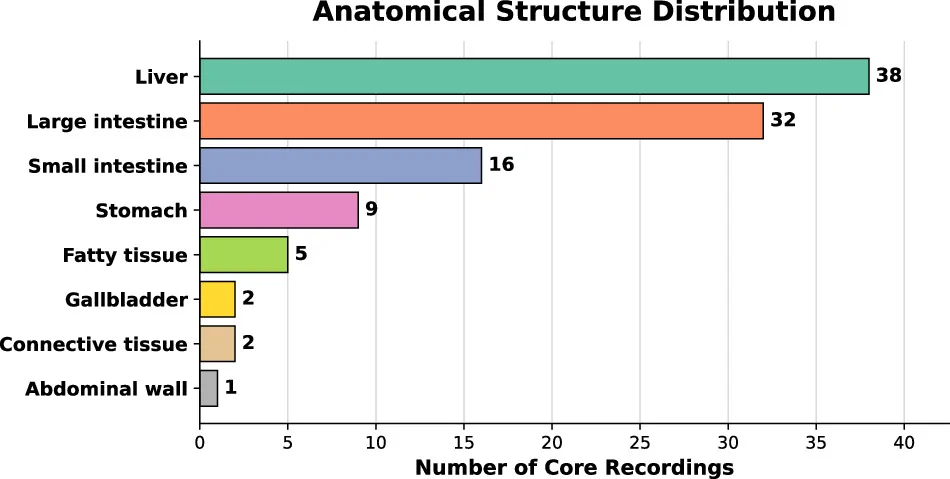
Distribution of the anatomical structures manipulated across the core sessions. Sessions that involved more than one structure contribute to each relevant category.

A surgeon operating the *da Vinci* system was asked to manipulate tissues in the abdomen from multiple viewing angles. The distribution of the anatomical structures manipulated, tabulated per session in info.csv, is shown in Fig. 3. These manipulations consisted primarily of pushing and pulling motions, repeated several times over the course of each session. Each clip followed a consistent structure: with the scene static and the endoscope light off, the SLC acquired a point cloud representing the baseline geometry; next, the endoscope light was switched on and the surgeon performed a tissue manipulation; finally, with the scene static again, the endoscope light was turned off again and a second SLC point cloud was captured to represent the post-action state. This alternating cycle ensured paired visual-geometric records of tissue deformation. As previously mentioned, three types of session were recorded: **(i)** whole deformations, representing complete push/pull actions; **(ii)** incremental deformations, divided into smaller steps to capture intermediate tissue states; and **(iii)** moved-camera sessions, where manipulation was split before and after camera repositioning to assess reconstruction continuity for structures moving out of view.

### Ethics Statement

No animals were euthanised for the purposes of this study. The porcine cadavers were used immediately post-mortem, following euthanasia carried out as part of separate, prior experiments conducted on the same day at the Experimental Operating Room of the NCT Dresden under approval number TVV 43/2023 (Saxon State Directorate). Because this work used only post-mortem tissue and involved no procedures on living animals, it required no additional animals and no separate animal-experiment approval. All animals were euthanised at the conclusion of those prior procedures while still under deep anesthesia with the intravenous injection of a lethal dose of potassium chloride, in accordance with the approved protocols.

### Postprocessing

Following data collection, we first checked the individual sessions for camera drift and that all data streams had been recorded correctly. Each session was then manually reviewed and sorted into one of three groups: a high-quality *core* set, an *ambiguous* set of lower or uncertain quality, and sessions that were discarded entirely. This was done to ensure that only reliable, high-quality sessions populated the core set used for evaluation, while still releasing the ambiguous sessions for any value they may hold.

A session was excluded from the core set, but retained in the ambiguous set, if any of the following applied:

- **Sensor movement**: the SLC was moved at any point, or the endoscope was moved during a static-camera session.
- **Endoscope light on during SLC capture**: the endoscope light was on while the SLC was capturing, which corrupts the SLC estimate of the scene geometry.
- **Motion during a static capture**: the tissue or surgical instruments moved while they were meant to remain static during an SLC capture.
- **Dark point cloud**: the SLC point cloud was too dark for its structures to be visually distinguished.
- **Missing capture**: one of the two SLC point clouds was missing from a whole-movement session.

A session was discarded entirely if any of the following applied:

- **No endoscope data**: the endoscope stream was missing in its entirety.
- **No SLC data**: the structured-light data were missing in their entirety.
- **False start**: the session was recorded accidentally and contained no intended manipulation.
- **Systematic point-cloud artefacts**: the point clouds were systematically choppy or patchy from physical disturbance of the SLC, such as air movement from a person walking past making it wobble slightly.

The retained *rosbag* files were decomposed then into files and folders consisting of individual data types (e.g. point clouds, images).

**Fig. 4:**
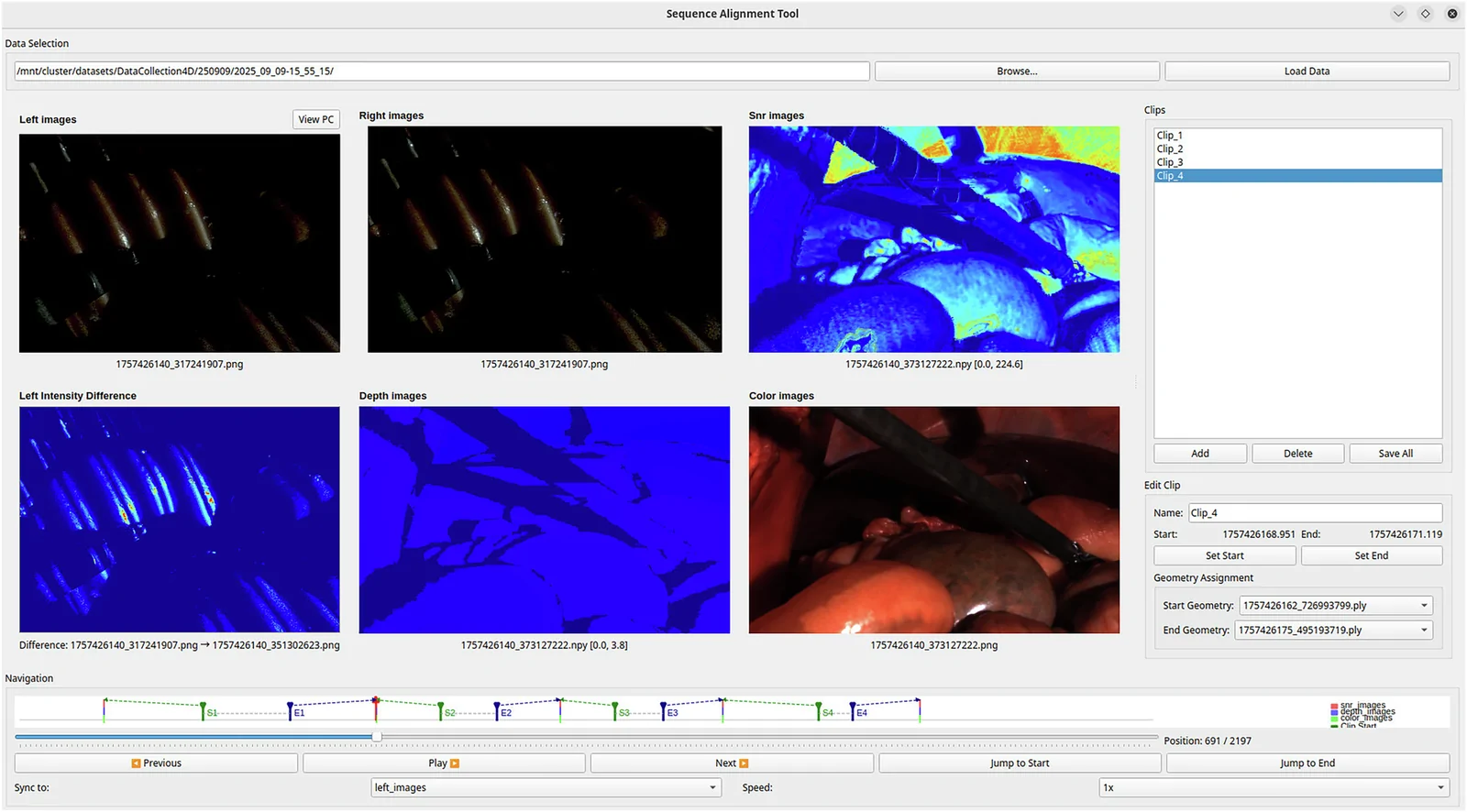
Sequence Alignment Tool used for creating clips.json files. After a session is loaded, the synchronised streams are shown: the left and right endoscope images, the SLC SNR, depth, and colour images, and a left intensity-difference image highlighting motion. Using the navigation timeline, the user marks the start and end frame of each clip (*Set Start*, *Set End*) and assigns the corresponding start and end SLC point clouds (*Geometry Assignment*).

**Fig. 5:**
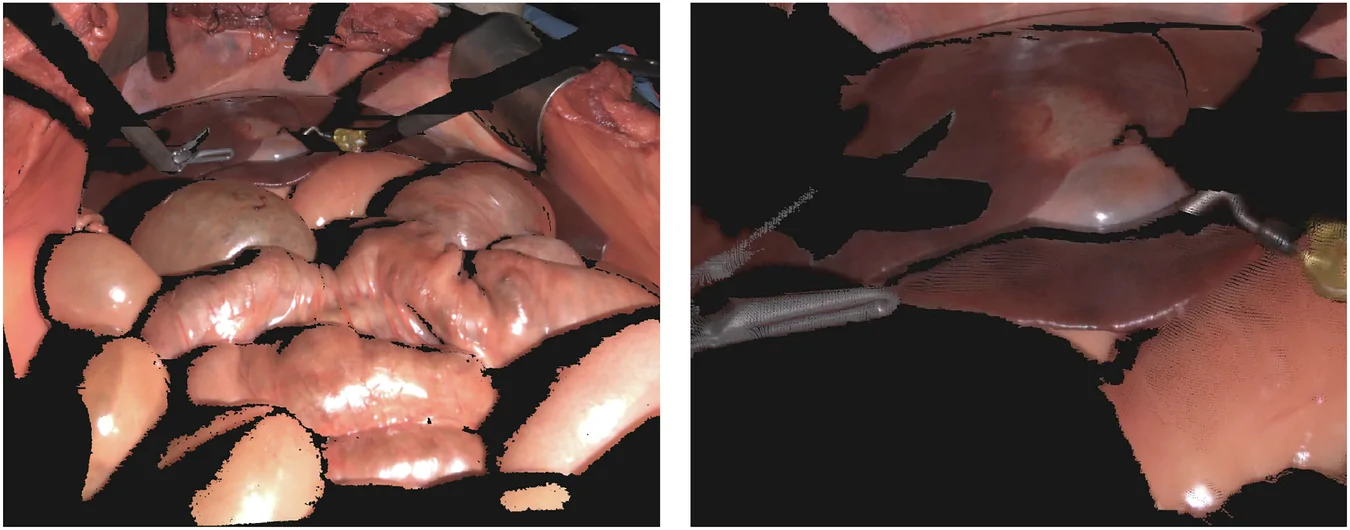
Point cloud acquired with the SLC rendered from the perspective of the SLC (left) and the endoscope camera after PnP-RANSAC (right).

**Fig. 6:**
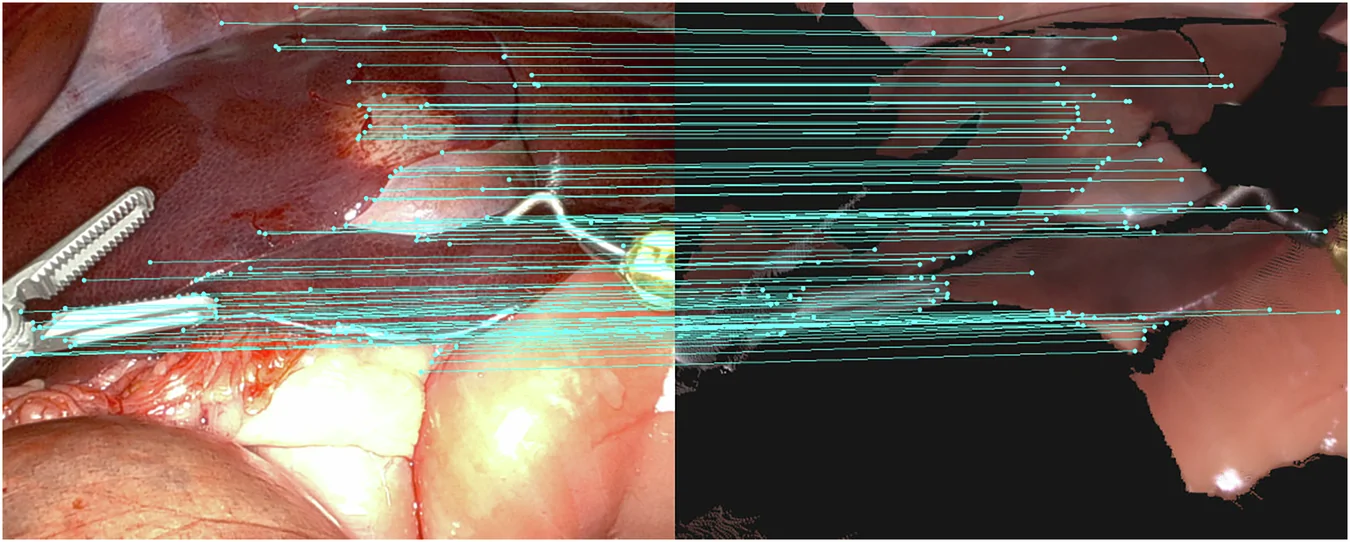
Visualization of alignment with feature matching (LightGlue).

**Fig. 7:**
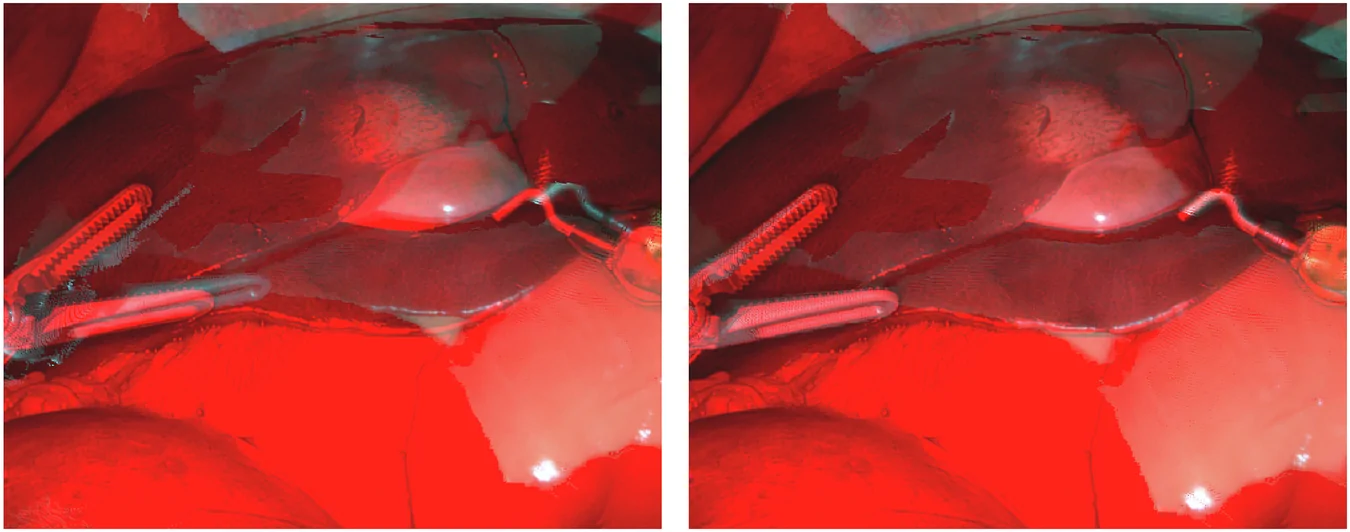
Red-cyan anaglyphs of the left endoscope image (red) overlaid with the SLC point-cloud rendering (cyan), using the nominal pose (left) and the PnP-RANSAC refined pose (right). Misalignment shows as doubled, offset structures, which largely disappear after refinement.

**Fig. 8:**
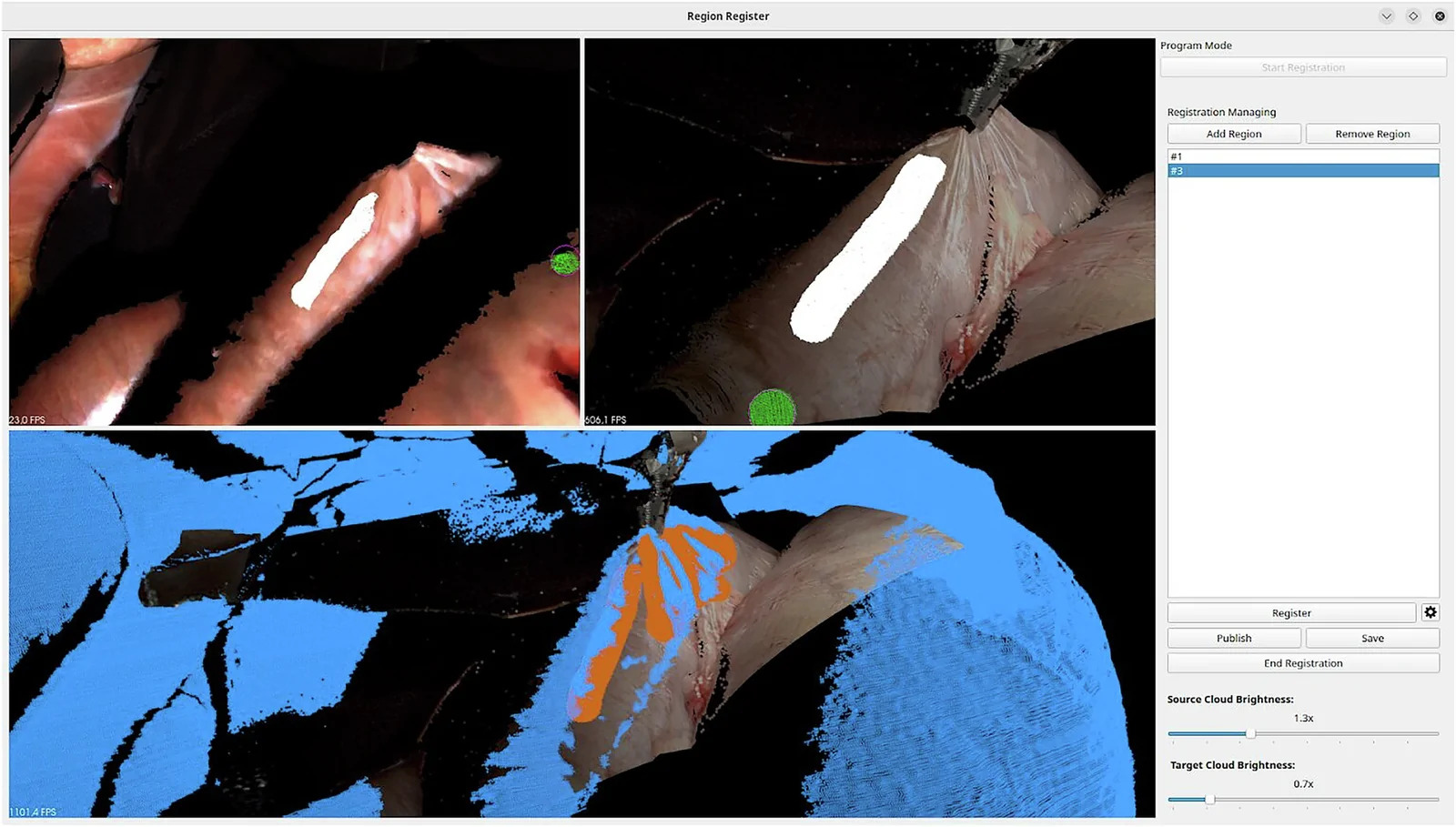
Example of the semi-automatic region-based ICP refinement used when standard ICP failed to produce a reliable alignment.

During the different recorded clips, before each tissue manipulation, there was a period for which the instruments and tissue were static due to preparing for the manipulation. Additionally, at the end of each tissue manipulation, the scene was not completely static once the instruments stopped moving because the tissues’ motion continued for a short time longer (through momentum or sliding). In order for the clips to comprise only the time when the tissues were deforming, a custom graphical tool (Fig. 4) was used to annotate the precise start and end points of each clip between successive SLC captures. This was based on visually identifying from the endoscope images the timepoints at which the tissue started and ended moving. To make these timepoints easier to identify, the tool displays a left intensity-difference image that highlights regions of inter-frame motion, alongside the synchronised endoscope stereo stream and the SLC colour, depth, and SNR images. Within the same tool, each clip was also linked to its start and end SLC point clouds. Although hand-eye calibration provided approximate pose alignment, residual errors were evident: rendered point clouds from the tracked endoscope perspective did not perfectly coincide with the endoscopic images. To mitigate this, point clouds were reprojected into the camera frame (Fig. 5) and aligned using feature detection and matching. A corrective transformation was then estimated with PnP+RANSAC^35^, using LightGlue^36^ as the primary matching algorithm (Figs. 6 and 7). In cases where automatic matching failed, correspondences were manually specified. The number of correspondences passed to the solver ranged from the minimum of four to many more, most commonly several tens. The pose was computed with OpenCV’s solvePnPRansac using the SOLVEPNP_ITERATIVE flag, which refines the pose by Levenberg-Marquardt minimisation of the reprojection error on the consensus inlier set. To further refine the poses, Iterative Closest Points (ICP) was carried out between the start and end SLC pointclouds and their stereo depth -derived counterparts. Some ICP registrations did not, however, obtain plausible alignments. As the endoscope camera was almost exclusively static during acquisition in different sessions, each alignment was visually inspected, and the single most fitting alignment was used as the curated pose for all static periods of that session, that is, for every clip and for both its start and end structured-light captures. For clips where no good alignment was found, a semi-automatic region-based ICP method^37^ with manual input was used to obtain higher quality registrations (see Fig. 8). Here, the stereo depth-derived and SLC point clouds are shown side by side and the user paints a few corresponding regions (typically three) onto matching anatomy. These regions are passed to a weighted ICP that weights the matched points heavily while the rest of each cloud still contributes, anchoring the alignment on reliable correspondences and avoiding the local minima that fully automatic ICP fell into. We refer to poses which were ultimately retained as ‘curated’ poses. Moved-camera clips consist of two static halves separated by a single repositioning. The start and end poses of each half are obtained through the same curation process as for static clips, while the nominal tracked poses are retained only for the intervening moving portion, where no SLC capture is available. No re-calibration or re-registration is performed after repositioning. The hand-eye calibration is carried out once during setup, and consistency across viewpoints comes from the per-half curation against the SLC point clouds rather than from the tracking.

Instrument segmentations were also created. For the endoscopic images, segmentations were obtained semi-automatically with the *Segment and Track Anything*^38^ and *SAM2*^39^ frameworks. Instruments were prompted with click points and the resulting masks propagated through each clip. We then reviewed every clip as a video with the masks overlaid to verify correctness, correcting any errors by adding prompts on the affected frame and re-propagating. For the SLC data, instrument segmentations were created manually for the start and end captures and are stored in each clip’s zivid_masks/ folder. IGEV++^40,41^ was used to create depth maps for all of the stereo endoscope image pairs. While all the original data are available at original resolution, these data are all scaled down to a resolution of (640 × 512) for straightforward compatibility with the methods which are evaluated downstream.

Prior to filtering out sessions which were not of high quality, there were 150 sessions collected altogether. After filtering, 98 sessions remained. A summary of the totals can be seen in Table 1. Save for the clearly unusable sessions, we are also releasing 30 ambiguous quality sessions in case they still provide some value, but without the substantial post-processing that we performed on the high quality sessions. By default, we intend that the *core* 98 sessions are used for evaluation.

**Table 1:** Summary of Session Counts by Specimen.

Specimen	Pre-Filter	Retained			
	Prior to Filter	Total	(i) Whole	(ii) Incremental	(iii) Moved Camera
0	24	0	0	0	0
1	37	16	9	7	0
2	14	14	7	5	2
3	19	15	8	6	1
4	33	32	15	12	5
5	23	21	10	9	2
**TOTAL**	**150**	**98**	**49**	**39**	**10**

For *Specimen 5*, the left and right images streams were mistakenly swapped during camera calibration, hand-eye calibration and all subsequent sessions. This was corrected in post-processing and all data can be used as for other specimens with the exception of the transforms in the tf/ folder (see below), which represent the recorded poses of the **right** endoscope camera.

## Data Records

The D4D dataset has been deposited in the OPARA repository at Technische Universität Dresden^42^. The dataset is organised in a three-level hierarchy. Each *specimen* is an individual porcine cadaver. Each specimen contains one or more *sessions*, which are continuous recordings named by their date and time. Each session contains one or more *clips*, the individual tissue manipulations that form the unit of evaluation. At the top level, there are specimen folders relating to each pig, each named in the order which data were collected (i.e., specimen_0, specimen_1, …). Each specimen folder contains multiple session folders, named by their recording date and time (e.g., 2025_03_06-16_49_40/).

Within each session folder, the following subdirectories and files are provided:

- camera_info/: camera calibration YAML files for endoscope and SLC;
- clips/: collection of clips, each stored as a separate Clip_X subfolder (see below);
- clips.json: metadata describing clip boundaries and timing;
- color_images/: RGB frames from the SLC (PNG, timestamped);
- depth_images/: depth maps captured by the SLC, timestamped;
- left_images/, right_images/: raw stereo image pairs prior to rectification;
- masks/: mask outputs obtained from SAM-Track^38^ and SAM2^39^;
- pointcloud/: 3D point clouds obtained from the SLC;
- snr_images/: signal-to-noise ratio maps associated with point cloud obtained from the SLC;
- tf/: transforms, timestamped.

Within each clips/ directory, individual clips are stored as Clip_X folders. Each clip aggregates the visual streams and geometric captures associated with one manipulation, along with derived products used for downstream reconstruction benchmarking:

- pose_bounds.npy: per-clip pose bounds (identity for static camera, nominal tracked poses for moved camera);
- curated_camera_pose_{start,end}.txt: curated camera poses for the start and end SLC captures;
- left_images_rect_masks/: per-frame binary masks (filenames are timestamps);
- left_images_rect/, right_images_rect/: rectified left/right images (PNG, timestamped);
- stereo_depth/: per-frame stereo depth (NumPy .npy, timestamped);
- zivid_images/: SLC colour images for the start and end captures (start.png, end.png);
- zivid_masks/: manually created binary instrument masks for the SLC colour images at the start and end captures (start_mask.png, end_mask.png);
- Clip_X.mp4: a preview video of the clip;
- camera_info/: resized camera calibration YAMLs for 640 × 512 resolution.

The session-level left_images/ and right_images/ folders hold the full, unrectified stereo streams for the entire session, whereas each clip’s left_images_rect/ and right_images_rect/ folders hold the rectified images for that clip only. All rectified images, masks, and depth maps are provided at 640 × 512 resolution to facilitate consistent benchmarking across reconstruction methods. Through an oversight, the rectified instrument masks of eleven clips were released at the unrectified 894 × 714 resolution instead. The accompanying d4d library resizes any rectified mask that is not already 640 × 512 when loading, so users of that interface are unaffected. This hierarchical structure (specimen_*/<date_time>/{clips/, camera_info/, ...}) mirrors the chronological collection of sessions and ensures reproducible linkage between the raw sensory data, per-clip reconstructions, and calibration metadata.

Lastly, further details, such as the tissue type which is manipulated in each session, is mentioned in info.csv. Whether a session features camera movement is indicated in session_details.txt.

## Technical Validation

**Fig. 9:**
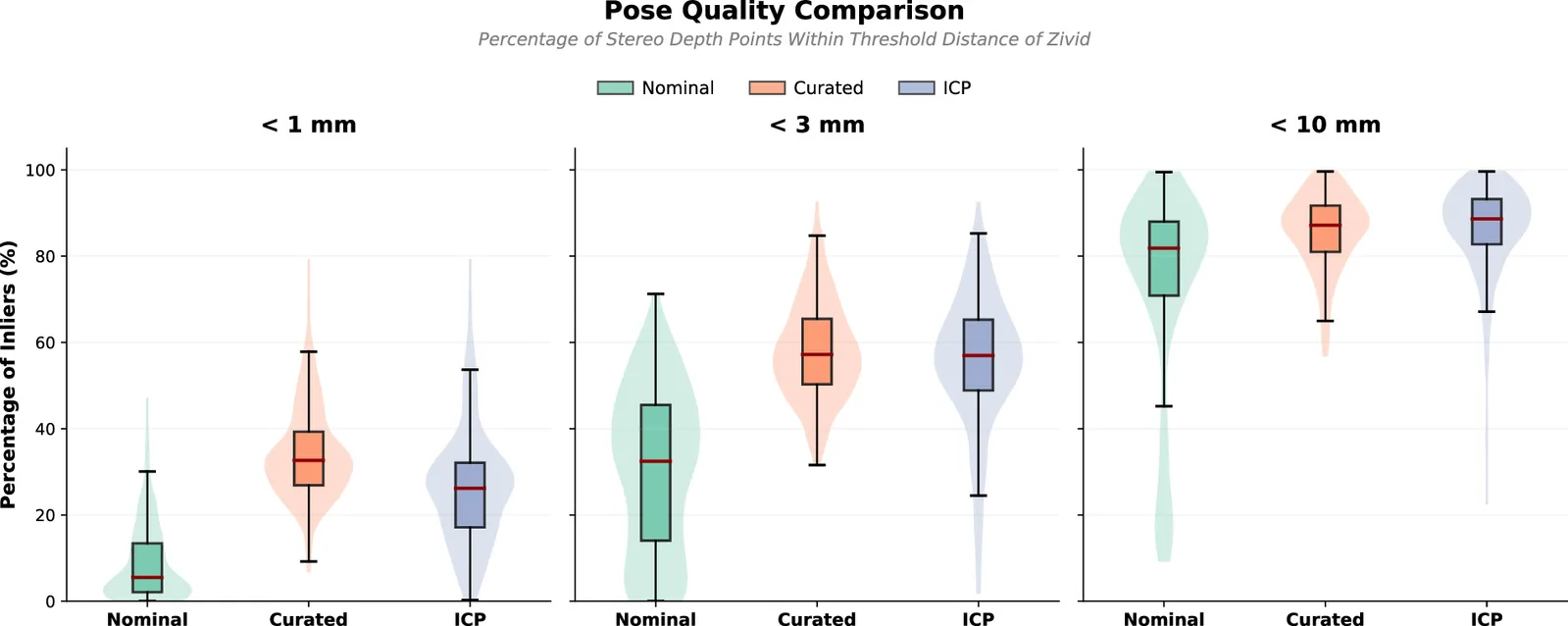
Comparison of pose quality across the Nominal, Curated, and ICP sets. The box plots summarize the distribution of nearest-neighbour distances between points in the Stereo Depth point cloud and their closest corresponding points in the SLC point cloud, illustrating the relative accuracy of the poses used in each set.

**Fig. 10:**
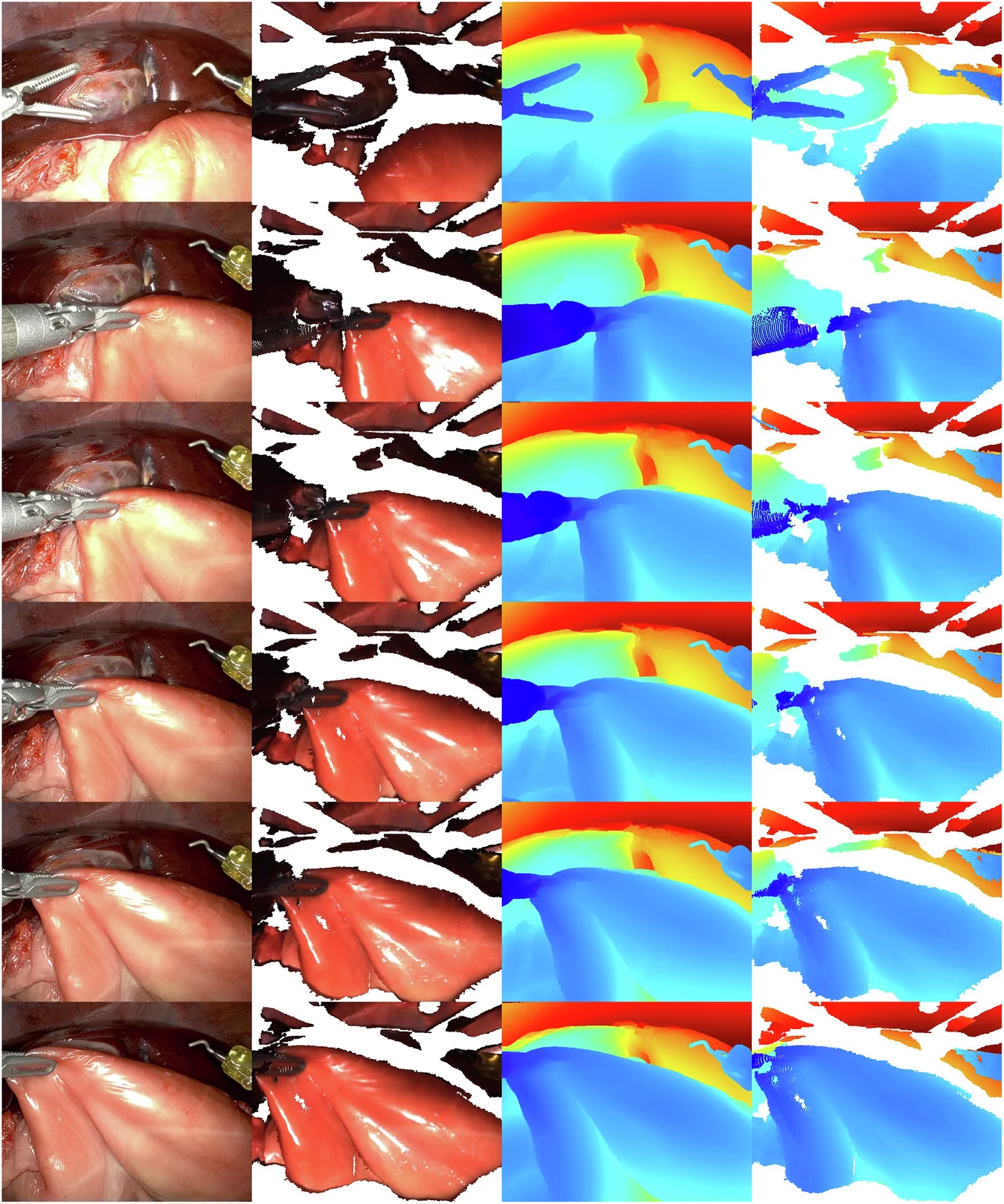
Overview of visual data associated with Clip 1 and the subsequent four clips for session *2025_09_09-15_40_48* of *Specimen 5*. For each clip (from top to bottom: the initial state of Clip 1 followed by the end states of the next four clips), the figure displays: the left endoscopic image, the RGB rendering produced from the SLC point cloud using the curated pose, the stereo depth map, and the SLC point-cloud depth image generated from the same curated pose. This layout illustrates the visual consistency and depth alignment achieved through the curated registration process.

**Fig. 11:**
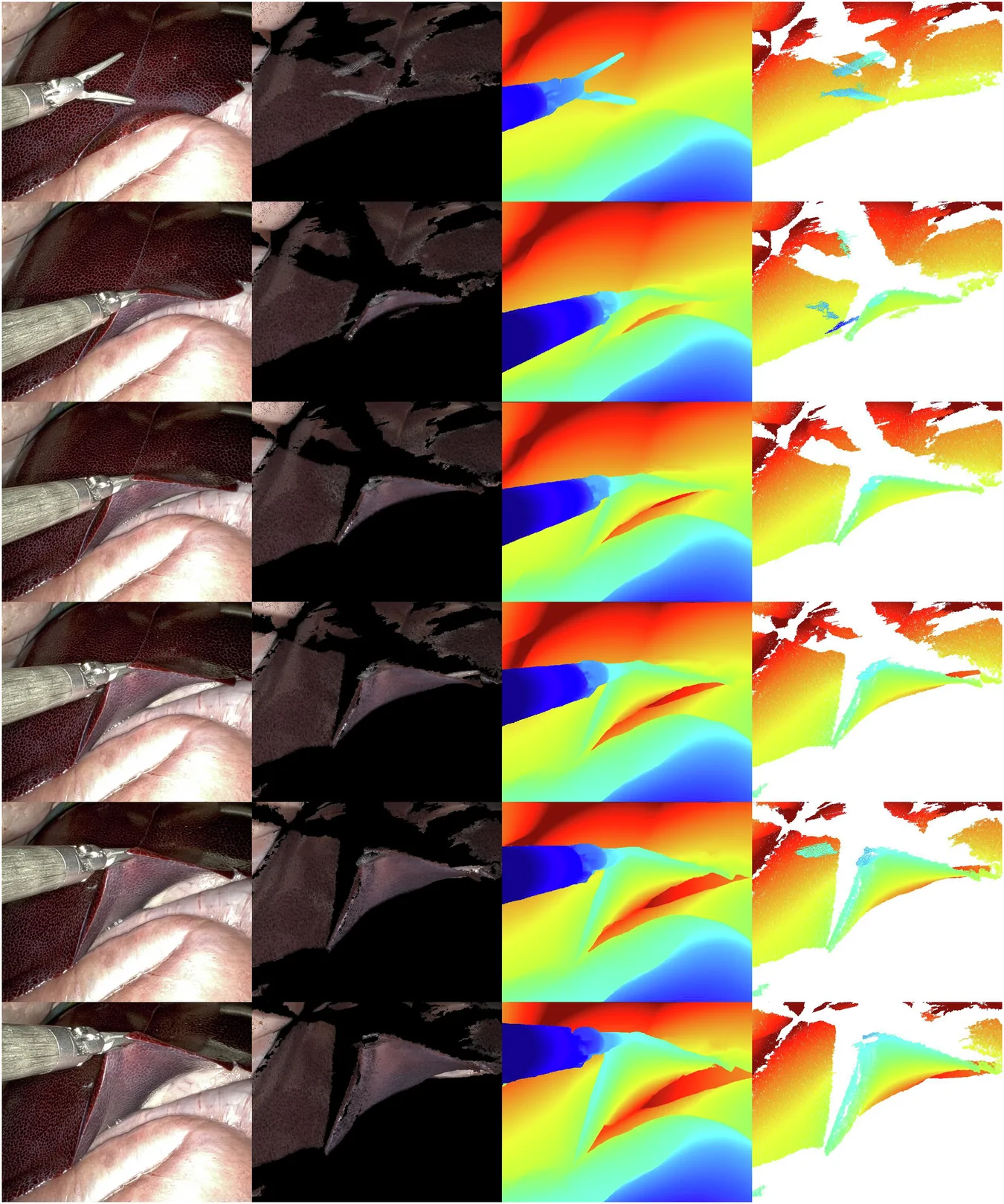
Worst-case alignment example, from session *2025_05_08-14_34_16* of *Specimen 3*. Columns and rows follow Fig. 10. The bottom-row Clip 5 end state has the maximum per-state median nearest-neighbour distance in the dataset (7.30 mm, against a dataset median of 2.15 mm in Table 2).

**Fig. 12:**
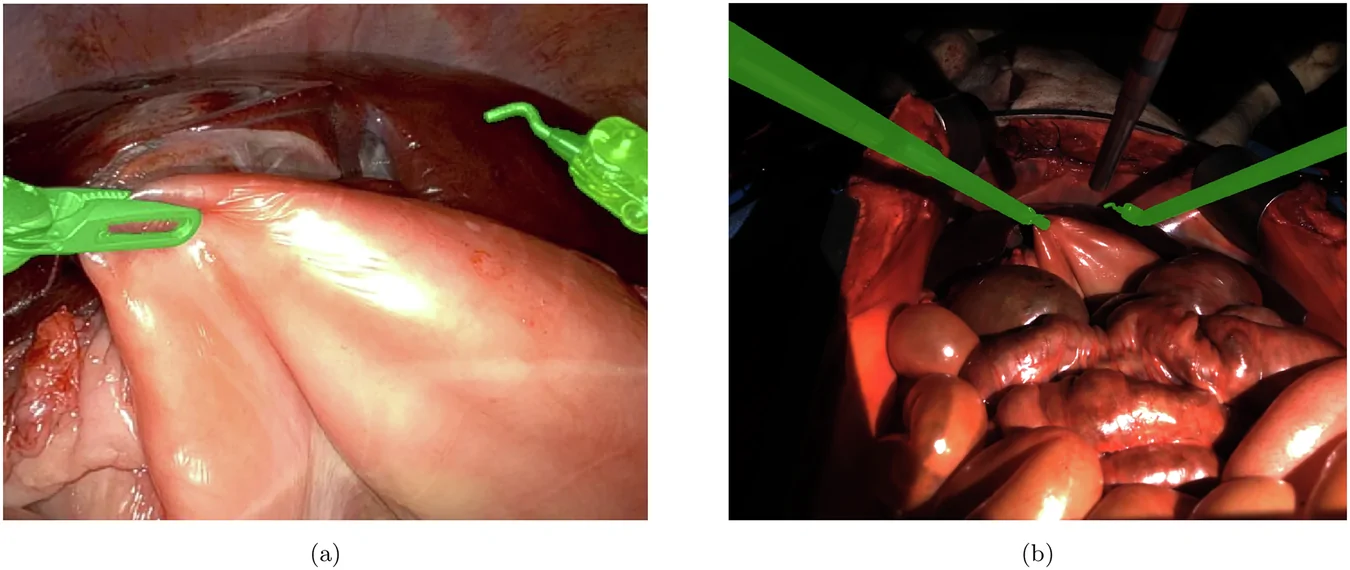
Session *2025_09_09-15_40_48* of *Specimen 5*, Clip 5 start (i.e., Clip 4 end) images with respective mask overlays: (**a**) Left endoscope view, (**b**) SLC view.

To illustrate the quality of the poses obtained through the curation process (composed of hand-selected ICP-derived and region-based registration poses), we report statistics describing the distance from each point in the Stereo Depth point cloud to its nearest neighbour in the SLC point cloud. These can be found in Table 2, with accompanying box-plots in Fig. 9. While the ICP registration has more inliers altogether when the threshold is set to 10mm, this changes for 3 mm and 1 mm thresholds where the curated poses deliver a greater proportion of inliers. This reflects the fact that corresponding points and surfaces between the two pointclouds most tightly align with the curated set of poses. This is further illustrated when comparing the RGB and depth images for the left endoscope images to their equivalents rendered from curated poses using the SLC pointclouds, as can be seen in Fig. 10. To show the magnitude of errors occurring in practice, Fig. 11 presents the worst-case session in the dataset, in which the largest per-state nearest-neighbour distance reaches 7.30 mm. Examples of the accompanying segmentation masks can be seen in Fig. 12.

**Table 2:** Summary statistics of point-to-point distances from points in the Stereo Depth-derived point cloud to the SLC point cloud after transformation with the Nominal, Curated, and ICP pose sets (N = 540 point clouds).

Metric / Set	Mean	Median	Std	Min	Max
**Median Dist (mm) - Nominal**	5.63	4.25	3.51	1.10	15.25
**Median Dist (mm) - Curated**	2.37	2.15	1.20	0.52	7.26
**Median Dist (mm) - ICP**	2.70	2.32	1.69	0.52	14.11
**Inliers < 1mm (%) - Nominal**	8.60	5.48	8.40	0.00	46.98
**Inliers < 1mm (%) - Curated**	33.90	32.62	10.38	6.84	79.11
**Inliers < 1mm (%) - ICP**	26.02	26.16	12.85	0.26	79.13
**Inliers < 3mm (%) - Nominal**	30.65	32.46	18.46	0.00	71.22
**Inliers < 3mm (%) - Curated**	57.89	57.22	11.19	31.58	92.45
**Inliers < 3mm (%) - ICP**	55.74	56.95	15.34	1.92	92.42
**Inliers < 10mm (%) - Nominal**	73.56	81.86	23.74	9.33	99.47
**Inliers < 10mm (%) - Curated**	85.71	87.16	8.63	56.91	99.62
**Inliers < 10mm (%) - ICP**	86.68	88.63	10.15	22.64	99.64

Each metric is first computed per point-cloud pair. For a given pair, the distance metric is the median nearest-neighbour distance from the stereo-depth cloud to the SLC cloud, and each inlier metric is the percentage of stereo-depth points whose nearest-neighbour distance falls below the stated threshold. The Mean, Median, Std, Min, and Max columns then summarise the distribution of these per-pair values across all 540 pairs.

**Fig. 13:**
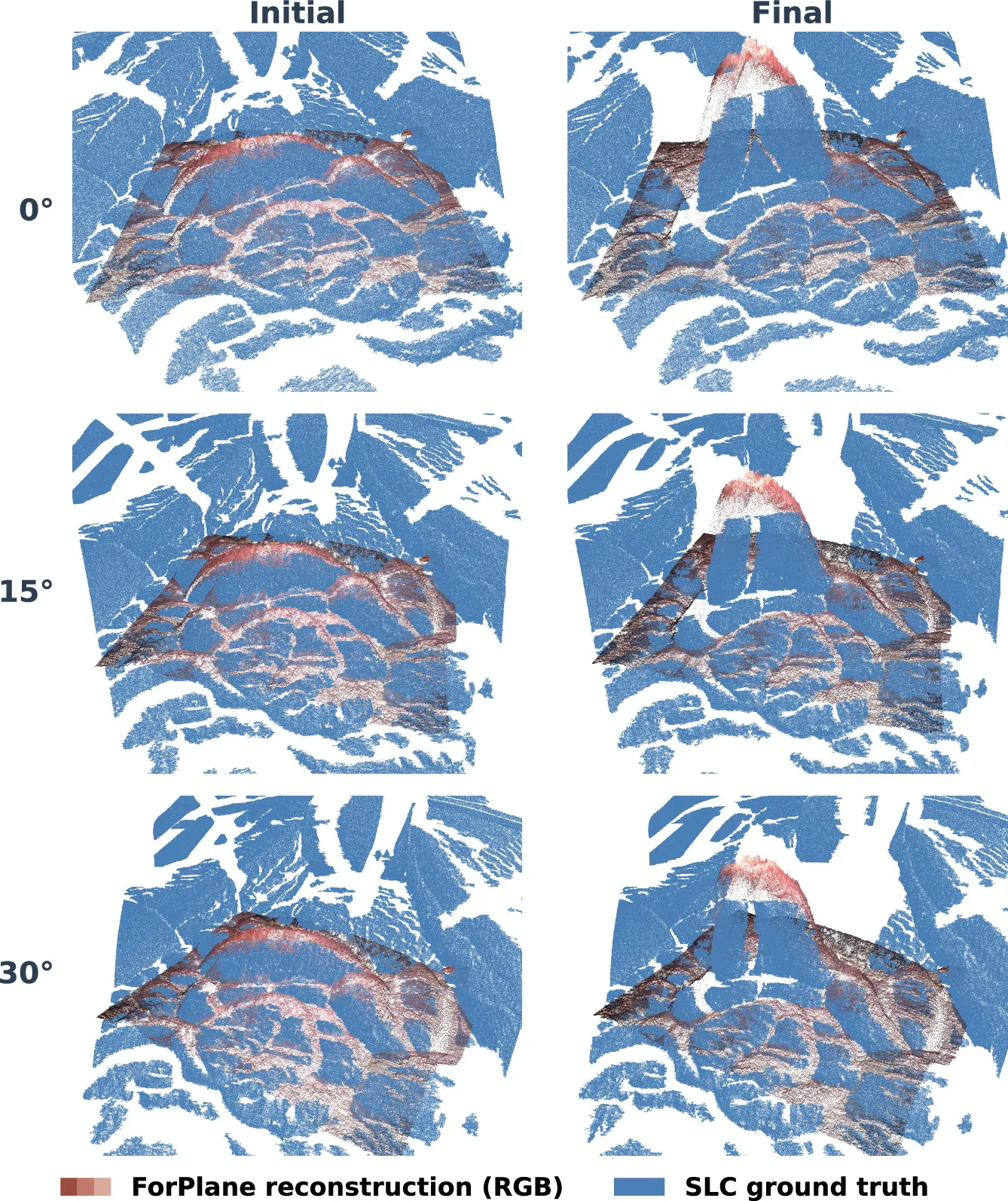
ForPlane^20^ reconstruction (in its reconstructed RGB colour) overlaid with the SLC ground-truth point cloud (blue) for Clip 1 of session *2025_03_06-16_49_40* (Specimen 1). The columns are the initial and final states. The rows are the scene rotated about the camera-to-reconstruction axis in 15° steps (0°, 15°, 30°).

As we intend for the dataset to be used to evaluate 4D Non-Rigid reconstruction in minimally-invasive abdominal surgery, we furthermore show the results of 3D reconstruction for the ForPlane Method^20^ on one of our clips. To demonstrate how the dataset is used to assess such reconstructions, the point clouds extracted for the initial and final states are overlaid with the corresponding SLC ground truth and shown from several viewpoints obtained by rotating the scene about the axis joining the endoscope camera centre to the centroid of the reconstruction (Fig. 13). The rotated views let the agreement between the reconstruction and the ground truth be appreciated from directions other than the original endoscope view.

## Usage Notes

This dataset supports research on non-rigid 3D/4D reconstruction, SLAM and depth estimation in minimally invasive surgery. Use rectified images, masks, and stereo depth in clips/, intrinsics in camera_info/, and curated_camera_pose_{start,end}.txt to align reconstructions to the SLC start/end point clouds. Evaluate geometry by comparing to SLC point clouds and optionally assess photometric quality of view-synthesis with PSNR/SSIM/LPIPS on non-masked pixels. Minor residual pose misalignments may remain despite the pose refinement steps undertaken.

*Whole* clips assess end-to-end robustness, *Incremental* clips probe deformation magnitude and enable a more granular study of tissue deformation, and *Moved-camera* clips test handling of out-of-view deformation and camera movement. While the *Moved-camera* clips test the reconstruction of out-of-view deformation in a more significant manner, all clips in fact possess out-of-view deformation whose evaluation this dataset also aims to enable. The ideal ground truth for this assessment would be to know correspondences between the points in the SLC pointclouds and points in the reconstructed pointclouds derived from the clips themselves - this is in practice, however, difficult to achieve. A lower-hanging fruit would be to evaluate simply what lies behind masked out tools, assuming that the respective tools have moved and what lies behind has already been observed during the clip.

If the evaluation protocol involves tuning hyperparameters (or training parameters) on the dataset prior to testing, we recommend a 5-fold leave-one-specimen-out cross-validation across the 98 *core* sessions of Specimens 1 to 5. In each fold, all sessions from four specimens are used for training and all sessions from the held-out specimen for testing, giving an approximate 4:1 train-to-test ratio. We do not provide a fixed train/test split file, so that the specimen-level folds can be reproduced directly from the specimen identifiers.

The data are usable with standard open-source Python tools and can be quickly interacted with through the repository below. The structured-light point clouds are stored as binary PLY files with coordinates in metres and per-point RGB colour, and load directly in Open3D and other tools that read PLY point clouds. Please cite this Data Descriptor when using the dataset.

## Data Availability

The D4D dataset is publicly available via OPARA at https://doi.org/10.25532/OPARA-1033. The dataset includes rectified stereo images, instrument masks, stereo depth maps, structured-light point clouds, curated camera poses, and camera intrinsics for 98 curated sessions across five porcine cadavers. The deposit also contains the 30 ambiguous-quality sessions described in Methods, provided as recorded and uncurated.

## Code availability

For ease of use, we are pairing the dataset with a repository which makes loading the data convenient and simple. The repository can be found at: https://github.com/reubendocea/d4d.
